# Metabolomic responses to the mechanical wounding of *Catharanthus roseus*’ upper leaves

**DOI:** 10.7717/peerj.14539

**Published:** 2023-03-20

**Authors:** Qi Chen, Yan Jin, Xiaorui Guo, Mingyuan Xu, Guanyun Wei, Xueyan Lu, Zhonghua Tang

**Affiliations:** 1 School of Life Sciences, Nantong Univesity, Nantong, Jiangsu, China; 2 Key Laboratory of Plant Ecology, Northeast Forestry University, Harbin, Heilongjiang, China; 3 First Affiliated Hospital, Heilongjiang University of Chinese Medicine, Harbin, Heilongjiang, China; 4 Northeast Agricultural University, Harbin, Heilongjiang, China

**Keywords:** *Catharanthus roseus*, Mechanical wounding, Metabolite, Source-sink

## Abstract

**Purpose:**

Plant secondary metabolites are used to treat various human diseases. However, it is difficult to produce a large number of specific metabolites, which largely limits their medicinal applications. Many methods, such as drought and nutrient application, have been used to induce the biosynthetic production of secondary metabolites. Among these secondary metabolite-inducing methods, mechanical wounding maintains the composition of secondary metabolites with little potential risk. However, the effects of mechanical stress have not been fully investigated, and thus this method remains widely unused.

**Methods:**

In this study, we used metabolomics to investigate the metabolites produced in the upper and lower leaves of* Catharanthus roseus* in response to mechanical wounding.

**Results:**

In the upper leaves, 13 different secondary metabolites (three terpenoid indole alkaloids and 10 phenolic compounds) were screened using an orthogonal partial least squares discriminant analysis (OPLS-DA) score plot. The mechanical wounding of different plant parts affected the production of secondary metabolites. Specifically, when lower leaves were mechanically wounded, the upper leaves became a strong source of resources. Conversely, when upper leaves were injured, the upper leaves themselves became a resource sink. Changes in the source-sink relationship reflected a new balance between resource tradeoff and the upregulation or downregulation of certain metabolic pathways.

**Conclusion:**

Our findings suggest that mechanical wounding to specific plant parts is a novel approach to increase the biosynthetic production of specific secondary metabolites. These results indicate the need for a reevaluation of production practices for secondary metabolites from select commercial plants.

## Introduction

Plant secondary metabolites represent important evolutionary adaptations and play an essential role in the response to environmental stress (Liu & Hao, 2018). These metabolites are also widely used to treat human diseases (Bills & Gloer, 2016). Most secondary metabolites can scavenge reactive oxygen species (ROS) and are anti-inflammatory, antibacterial, and antiviral (Lian et al., 2021). Among these metabolites, phenolic compounds (PCs) have particularly great medicinal value. Quercetin, for instance, plays an important antitumor role by reducing the drug resistance of tumor cells and inducing the apoptosis of tumor cells (Hashemzaei et al., 2017). Naringin inhibits the apoptosis of vascular endothelial cells and promotes intraosseous angiogenesis (Shangguan et al., 2017). Plasma-conjugated metabolites of orally administered water-dispersible hesperetin improve vasodilation in endothelial cells (Zhang et al., 2020). Other secondary metabolites such as terpenoid indole alkaloids (TIAs) have also been widely used in medicine. *Catharanthus roseus* (L.) G. Don (*C. roseus*) is an important medicinal plant in the investigation of TIAs. Vinblastine, an efficient inhibitor of microtubule polymerization, has been used to treat human neoplasms (Lee et al., 2016). Another TIA serpentine can effectively block the transmission of adrenergic nerve impulses, resulting in vasodilation, lower blood pressure, and a slower heart rate (Mukherjee et al., 2019). It has also been indicated that secondary metabolites can reduce blood fat, delay senility, and improve immunity (Mukherjee et al., 2019).

The synthesis of many secondary metabolites is regulated under stress conditions such as UV-B, wounding, drought, metal toxicity, and nutrient deprivation (Takshak and Agrawal, 2019; Blum, 2017; Erika, Jorge & Daniel, 2018). Mechanical wounding from rain, hail, wind, and herbivores are the most common types of damage that plants face. This can lead to nutrient loss in the damaged tissues, which increases the risk of pathogenic invasions. However, this crisis also results in the massive accumulation of secondary metabolites (Sun et al., 2020). Mechanical wounding is an ideal way to induce the production of secondary metabolites because it reduces the risk of other stresses that pollute secondary metabolites. Nevertheless, the changes involved in secondary metabolites under mechanical wounding stress have been rarely reported (Ibanez et al., 2019).

Metabolomics is a powerful approach used for discovering considerably different metabolites and a useful tool for pinpointing endpoint metabolic effects from external stimuli. It has been widely used for exploring the contents of metabolites, identifying key metabolites, and deciphering central metabolic pathways in plants (Jiang et al., 2019; Li et al., 2016). For example, GC-MS technology has been adopted to compare the medicinal activities of different tissues of *Zingiber mioga* and *Zinger roscoe*, suggesting that various structural parts of plants have different dietary usages (Soo et al., 2015). Wang et al. (2016) discovered that polyphenol accumulation and stress resistance preparation in cacao seed (*Theobroma cacao*) ripening occurred via the interplay of primary and secondary metabolites at the system level. *C. roseus*, originating from the coast of the Mediterranean, India, and tropical areas in America, has been extensively studied for its highly economic and pharmaceutical value (Liu et al., 2017; Moon, Mistry & Kim, 2017). The main metabolite TIAs of *C. roseus* are widely used to treat human diseases. It is considered a remarkable manufacturer of secondary metabolites, and more than 130 kinds of TIAs from the plant have already been described (Pham et al., 2020). Its biosynthesis pathway starts from the coupling of tryptamine and secologanin, then strictosidine is formed by strictosidine synthase (Pham et al., 2020). In the next steps, tabersonine and vindoline are synthesized separately by strictosidine *β*-glucosidase and deacetylvindoline acetyl CoA acetyltransferase (Singh et al., 2020). Finally, vindoline couples with catharanthine to form valuable vinblastine catalyzed by Peroxidase 1 (Singh et al., 2020). Many of these TIAs are natural anticancer agents, including loganin, catharanthine, serpentine, vindoline, vinblastine, and vincristine. Vinblastine is an efficient inhibitor of microtubule polymerization that is used to treat certain cancers such as Hodgkin’s disease, malignant lymphoma, and a wide variety of other human neoplasms (Mondal et al, 2019). In this study, metabolomics was used to investigate different responses to wounding and the time specificity of secondary metabolites in upper leaves. Our research provides basic data for the response of secondary metabolites to mechanical wounding.

## Materials & Methods

### Plant materials and treatment

**Figure 1:**
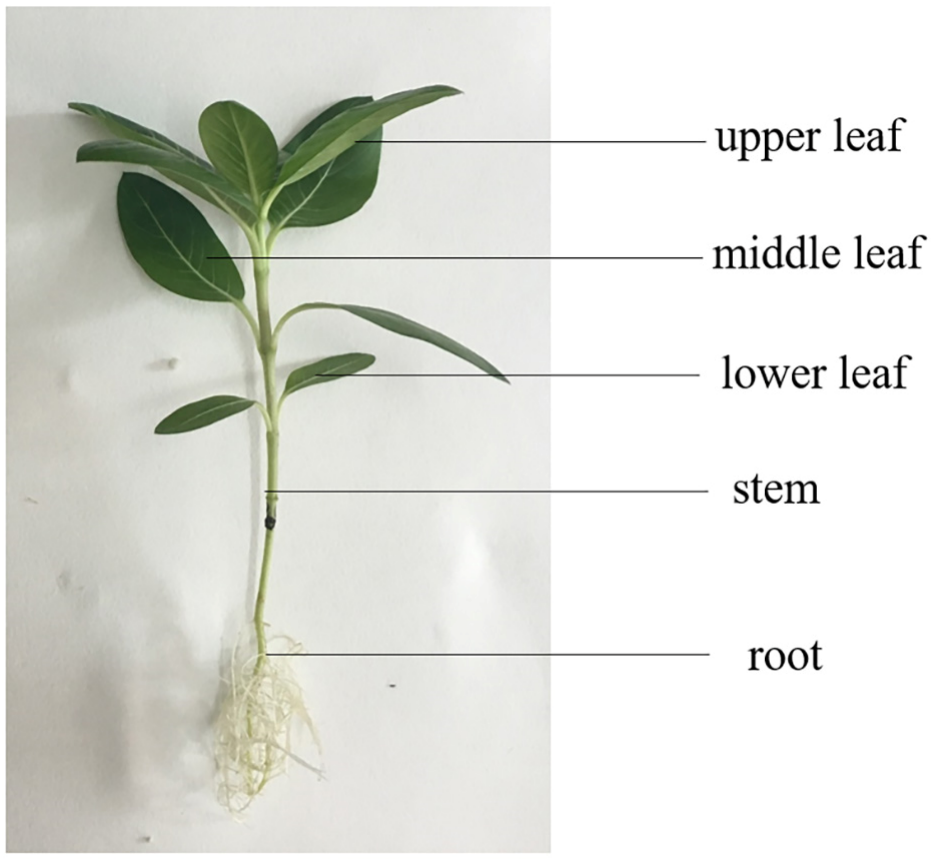
Leaf, stem, and root anatomy of *Catharanthus roseus*.

*C. roseus* seeds were sown in pots and grown in a climate chamber (S10H, Conviron, Winnipeg, Canada) with 14 h light (28 °C, irradiation of 450 µmol m^−2^ s^−1^) and 10 h dark (25 °C, without irradiation) regime at a humidity of 60%. *C. roseus* seeds were cultivated with hydroponics and irrigated with 1/2 strength Hoagland’s solution (pH 5.9−6.0). After 80 d, when seven to eight true leaves had developed, plants were randomly assigned to three groups with four plants in each group. They were subjected to either a sham procedure (the control group, CK), mechanical wounding in the upper leaf (the wounded upper leaf group, WUL), or mechanical wounding in the lower leaf (the wounded lower leaf group, WLL). Mechanical wounding was completed with the trim of 1/2 leaves. Different parts of *C. roseus* (upper leaf, middle leaf, lower leaf, stem, and root) were collected (Fig. 1). According to the experimental results, the detected parts of plants for the next stage of the experiment were obtained. Each group were detected at 0 h, 1 h, 3 h, and 5 h after wounding. Experiments were conducted for four replicates.

### Metabolite profiling

*C. roseus* samples were analyzed as previously described (Chen et al., 2017). Briefly, 60 ± 5 mg of plant tissue was gathered, mixed with 360 µL cold methanol and 40 µL 0.3 mg/mL 2-chlorophenylalanine, homogenized (Tissuelyser-192, Shanghai, China), and sonicated for 30 min, then 10,000 g for 10 min at 4 °C. For ultrasonication, 200 µL chloroform and 400 µL water were added to the sample. Samples were methoxyaminated and silylated after dying. After derivatization, samples were analyzed on the GC-MS (Agilent Corporation, Santa Clara, CA, USA). A nonpolar DB-5 capillary column was used for separation. The temperature program was 50−125 °C for 8 min, raised to 125−170 °C for 15 min, raised to 170−210 °C for 4 min, raised to 210−270 °C for 10 min, raised to 270−305 °C for 5 min, and maintained at 305 °C for 5 min. Injection and ion source temperatures were set at 260 °C and 230 °C, respectively. Electron impact ionization (−70 eV) proceeded at full scan mode (m/z 30 −600). The acquisition speed was 20 spectra/s, and mass spectrum (MS) data were analyzed by Chroma TOF software. The data set was normalized using the sum intensities of the peaks in each sample.

For the analyses of secondary metabolites, 1.0 g fresh tissues were mixed with 20 mL analytical grade absolute methanol and exposed to low-frequency ultrasonication (250 W, 40 kHz) for 40 min. After centrifugation at 7,104 g for 10 min, alkaloids were determined using HPLC-MS (Ultra-performance LC, Waters, Milford, MA, USA; MS, AB SCIEX, Framingham, MA, USA) with ACQUITY UPLC BEH C18 Column (1.7 µm, 2.1 mm ×50 mm). TIA content was measured based on the previously described method (Chen et al., 2017). Chromatographic analysis was performed on ACQUITY UPLC BEH C18 Column (1.7 µm, 2.1 mm × 50 mm). The standard solvent system was CH3CN/H_2_O and 0.05 mol/L ammonium acetate. Retention times were 3.49 min (serpentine), 2.91 min (tabersonine), 3.37 min (vindoline), 3.32 min (vinblastine), 2.81 min (catharanthine), and 0.76 min (loganin). Injection volume was 10 mL and flow rate was one mL/min. After 20 mL methanol extraction, targeted analysis of phenolic metabolites (PCs) was performed using a Waters ACQUITY UPLC system (Waters, Milford, MA, USA) coupled to a quadrupole time-of-flight (QTOF) mass spectrometer (XEVO G2 QTOF, Waters, Milford, MA, USA). The optimized chromatographic conditions were: A%, 0.05% formic acid water; B%, 0.05% formic acid acetonitrile; 120–1,200 m/z; positive ion scanning mode; and leucine enkephalin.

### Statistical analysis

Normalized data were imported into SIMCA-P software (version 13.0, http://www.umetrics.com/simca). A supervised orthogonal partial least squares discriminant analysis (OPLS-DA) was used to identify differential metabolites among the CK, WUL, and WLL groups. Metabolites with both multivariate and univariate statistical significances (VIP >1.0 and *p*-value <0.05) were extracted by OPLS-DA analysis. Differential metabolites were annotated using the KEGG database (http://www.kegg.jp/kegg/pathway.html) and MBRole 2.0 (http://csbg.cnb.csic.es/mbrole2/). The score of principal component “Q” (Q) was calculated using SPSS software (version 21.0; Chicago, IL, USA). Histograms and pathway maps were generated using GraphPad Prism (version 6.0; GraphPad Software Inc., La Jolla, CA, USA) and Visor (Microsoft, Redmond, WA, USA).

## Results

### Screening of plant detection tissues

**Figure 2:**
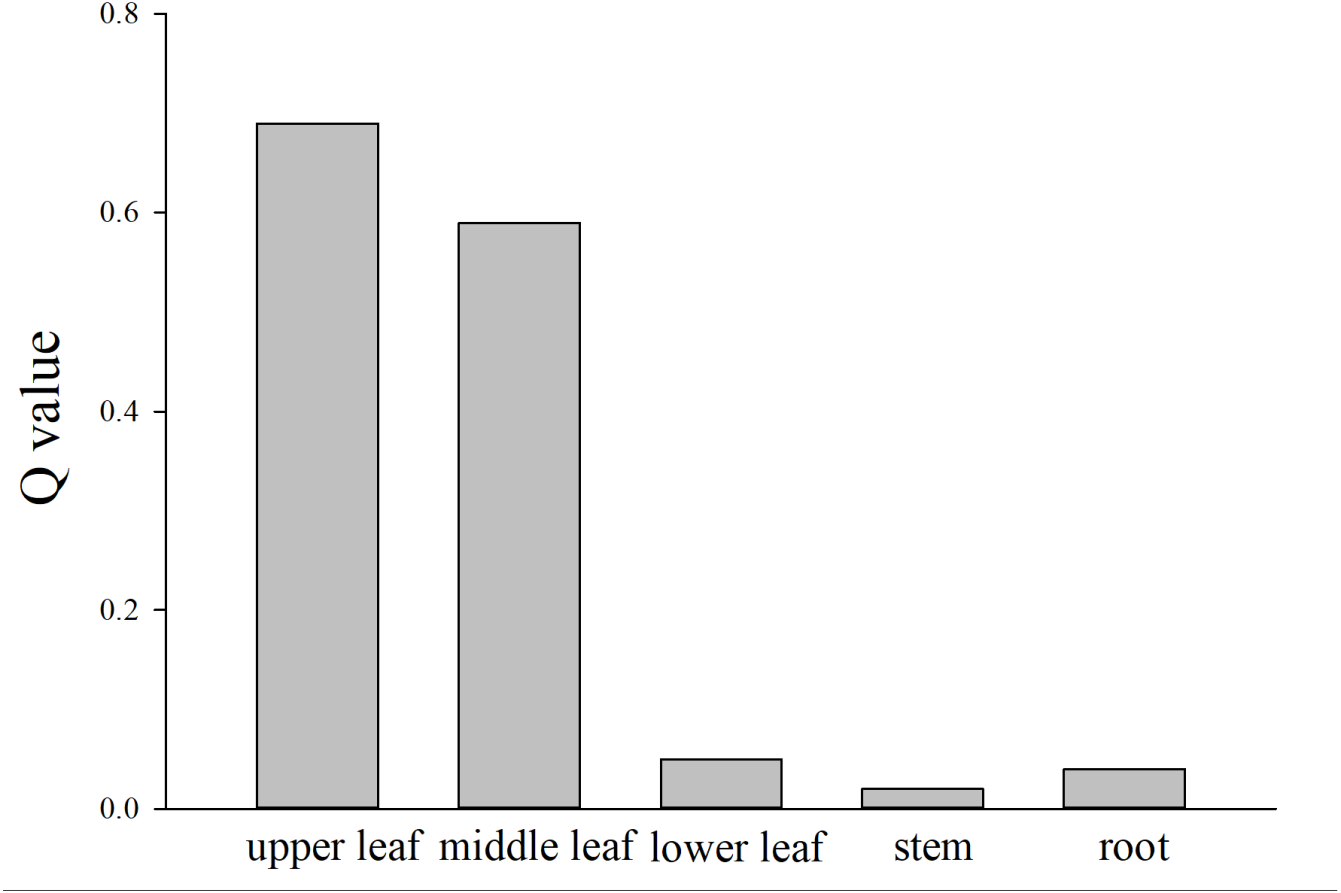
*Q* values of secondary metabolites in upper leaf, middle leaf, lower leaf, stem, root of *Catharanthus roseus*.

After screening, we calculated the *Q* values of secondary metabolites TIAs and PCs sampled from different parts of *C. roseus*. Stems and roots had robustly lower *Q* values than leaves. Upper, middle, and lower leaves also showed different *Q* values with upper leaves obtaining the most intense responses (Fig. 2). Therefore, upper leaves of *C. roseus* were collected for subsequent metabolomics experiments.

### Primary metabolites

**Figure 3:**
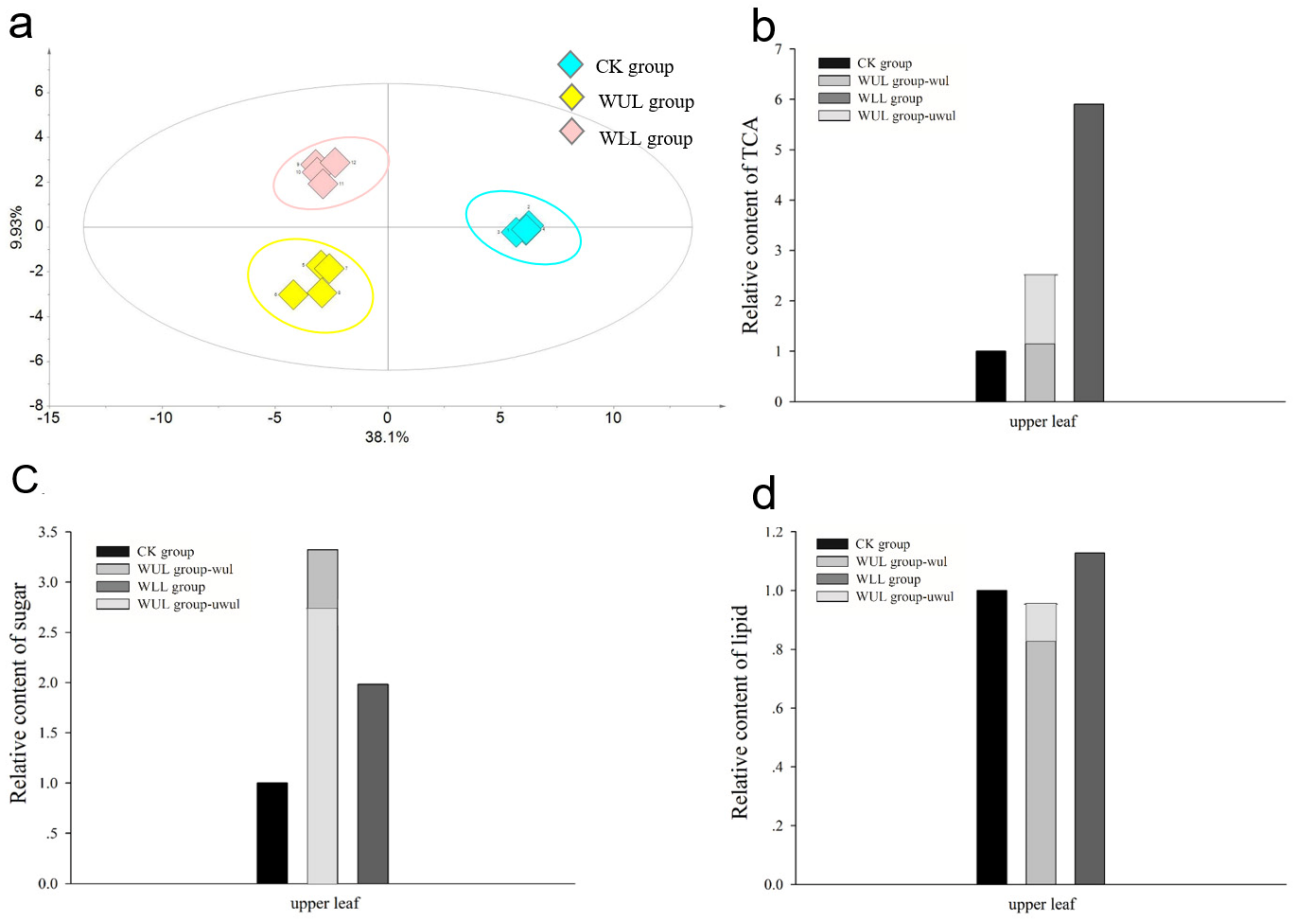
Changes primary metabolites. (A) OPLS-DA score plot of primary metabolisms. (B) Relative content of TCA component. (C) Relative content of sugar. (D) Relative content of lipid. CK, control group; WUL group, wounded upper leaf group; WLL group, wounded lower leaf group; WUL group-wul, wounded part in wounded upper leaf group; WUL group-uwul, unwounded part in wounded upper leaf group.

To compare the metabolic variations across the CK, WUL, and WLL groups, OPLS-DA was applied. The CK, WUL, and WLL groups were separated by PC1 (38.1%) and PC2 (9.9%) (Fig. 3A). Eleven significantly different metabolites (glucosamine, galactose, xyolopyranose, tagatose, fructofuranose, gentiobiose, fructose, galactitol, octadecanoic acid, hexadecanoic acid, and succinate) were screened from a total of 133 compounds by VIP >1 and a *p*-value <0.05 criteria (Table 1). These metabolites presented substantial differences in energy supply between the control and wounded groups. Our results suggest that TCA members and sugars significantly increased with mechanical wounding (Figs. 3B and 3C). On the other hand, lipid content was not robustly altered (Fig. 3D). These findings also indicate that TCA members and sugars were in great demand by plants under stress.

**Table 1:** List of significantly different metabolites.

	Significantly different metabolites	VIP	*p*-value
sugar	glucosamine	1.85	^*^
	galactose	1.49	^**^
	xylopyranose	1.17	^*^
	tagatose	1.16	^**^
	fructofuranose	1.06	^**^
	gentiobiose	1.06	^*^
	fructose	1.04	^*^
	galactitol	1.02	^**^
lipid	octadecanoic acid	1.21	^*^
	hexadecanoic acid	1.08	^*^
TCA component	succinate	1.02	^**^

**Notes.**

VIP, variable importance in the projection.

* *p*-value <0.05.

** *p*-value <0.01.

Data are summarized from three biological replicates.

### Metabolic fingerprint of TIAs and PCs

**Figure 4:**
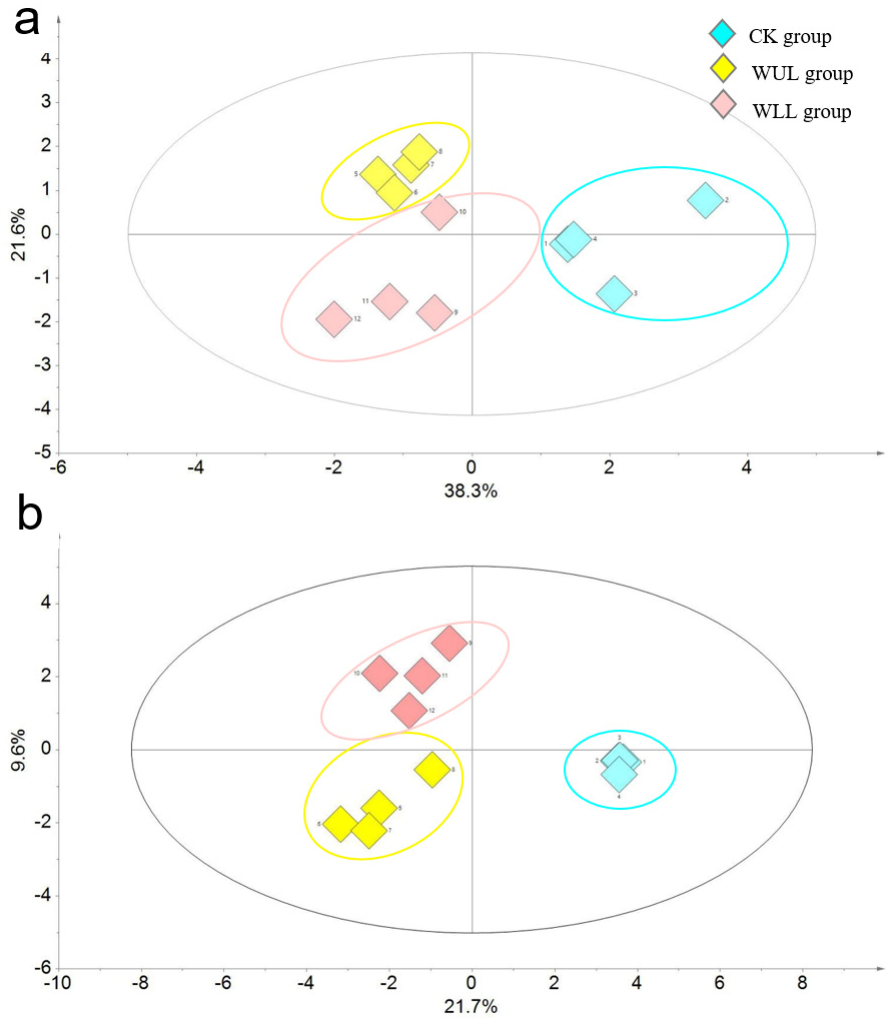
OPLS-DA score plot of secondary metabolites. (A) OPLS-DA score plot of TIAs. (B) OPLS-DA score plot of PCs. CK, control group; WUL group, wounded upper leaf group; WLL group, wounded lower leaf group.

**Figure 5:**
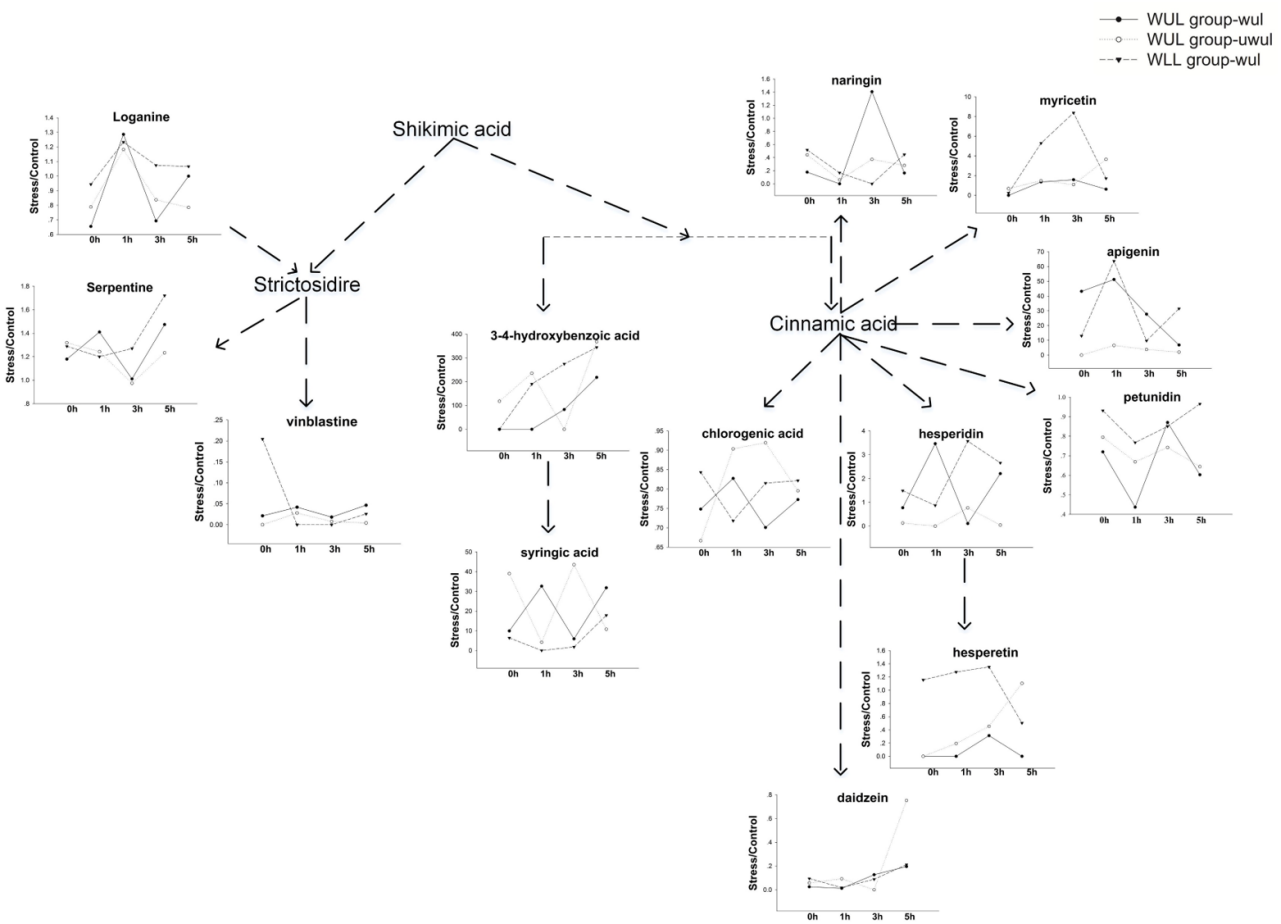
The network of altered metabolites after mechanical stress. WUL group-wul, wounded part of wounded upper leaf group; WUL group-uwul, unwounded part of wounded upper leaf group; WLL group-ul, the upper leaf of wounded lower leaf group.

The dynamic changes of secondary metabolites in the upper leaves of the control and wounded groups at 0 h, 1 h, 3 h, and 5 h after mechanical wounding were further investigated in detail. TIAs and PCs were separated from the control and wounded groups by OPLS-DA analysis (Fig. 4). The WUL and WLL groups were separated by 21.6% of PC2 in TIAs and 21.7% of PC1 in PCs (Fig. 4). Three different TIAs (vinblastine, loganin, and serpentine) and 10 different PCs (hesperetin, petunidin, daidzenin, chlorogenic acid, syringic acid, hesperidin, naringin, 3-4-hydroxybenzoic acid, and apigenin) were obtained using VIP values (VIP >1) and *p*-values (*p-value* <0.05) (Tables 2 and 3). The abundance of these 13 different secondary metabolites was significantly altered at different time points (Fig. 5). The metabolic fingerprint showed that mechanical wounding changed metabolic direction in a tissue-specific and time-dependent manner. Synthetic raw materials and energy from shikimic acid flowed into alkaloid and phenol metabolism, respectively (Fig. 5). Upstream precursor metabolites such as loganin, for instance, responded at 1 h with the most intense response in wounded leaves. Vinblastine, a downstream final product, was substantially reduced in the wounded group. Conversely, serpentine showed a continuously increasing trend, especially in the WLL group.

**Table 2:** The significantly different TIAs.

Significantly different metabolites	VIP	*p*-value
vinblastine	1.48	^*^
loganine	1.24	^*^
spertine	1.10	^**^

**Notes.**

VIP, variable importance in the projection.

* *p*-value <0.05.

** *p*-value <0.01.

Data are summarized from three biological replicates.

**Table 3:** List of significantly different PCs.

Significantly different metabolites	VIP	*p*-value
hesperetin	1.81	^**^
petunidin	1.54	^*^
daidzein	1.50	^*^
myricetin	1.37	^**^
chlorogenic acid	1.32	^**^
syringic acid	1.28	^*^
hesperidin	1.17	^*^
naringin	1.16	^**^
3-4-hydroxybenzoic acid	1.15	^*^
apigenin	1.02	^*^

**Notes.**

VIP, variable importance in the projection.

* *p*-value <0.05.

** *p*-value <0.01.

Data are summarized from three biological replicates.

The PC results showed that the relative contents of chlorogenic acid, petunidin, and daidzein remarkably decreased, suggesting that the synthesis of PCs was affected by mechanical wounding. Myricetin, apigenin, hesperidin, and hesperetin responded to mechanical wounding in the WLL group 3 h after wounding, indicating that these metabolites might participate in common defense reactions. In the WUL group, C6C3C6-structure PCs naringin and hesperidin responded rapidly to wounding. C6C1-structure PCs exhibited a different response to mechanical wounding. 3–4-hydroxybenzoic acid expression elevated hundreds of times at 1 h after wounding and continued to increase at later time points. By comparison, the activation of syringic acid was the opposite in wounded and unwounded leaves in the WUL group, indicating that syringic acid works differently.

## Discussion

Secondary metabolites not only play a key role in protecting plants against stress but are also integral in natural medicine (Rumzum et al., 2020). Many secondary metabolites that are used in healthcare are often in short supply (Bills & Gloer, 2016). Mechanical wounding can stimulate secondary metabolite accumulation and reduce potential hazards from other interference factors (Savatin et al., 2014). The types of secondary metabolites, as well as their abundance, can be affected by the specific part of plant leaf (*i.e.*, upper or lower) and the location of the mechanical wound. In this study, we employed high throughput methods to explore the response mechanisms of the various parts of *C*. *roseus*.

Primary metabolites of leaves responded to the mechanical wound. After mechanical wounding, 11 significantly different metabolites, including sugars, lipids, and TCA components, were screened from a total of 133 identified compounds. Among them, some metabolites demonstrated a clear link between mechanical stress and remodeling of the central metabolism. They increased the basic demands of the upper leaves in the wounded group. The function of sugars in this process was mainly to provide substrates for energy production and biosynthesis of secondary metabolites to increase plant resistance. It could also be used as a carbon intermediate in the metabolic cycle.

Besides primary metabolites, the contents of secondary metabolites TIAs, and PCs were also determined. TIAs and PCs are two branches of secondary metabolites that respond to mechanical stress according to their coordination or competition in trade-offs and distribution. In our study, three significantly different metabolites were obtained from the TIA pathway within the different leaf position treatments. Relative to the control group, the content of loganin and serpentine significantly increased following mechanical treatment. Serpentine was generally distributed in the root of *C. roseus* but largely accumulated in upper leaves after mechanical stress. The loganin-serpentine pathway was found to be stimulated by mechanical wounding, indicating that mechanical stress can improve the yield of some TIAs. Serpentine can combine with norepinephrine to reduce blood pressure (Francisco et al., 2013). Loganin is widely used in clinical medicine as an anticancer drug (Chen et al., 2022). Interestingly, the content of vinblastine also significantly change. They are the end product of the TIAs metabolic pathway. Vinblastine could effectively treat cancer. It was found could management of advanced angiosarcoma by the synergistic combination of Propranolol based metronomic chemotherapy (Pasquier et al., 2016). It is meaningful to affect the content of vinblastine by regulating TIAs metabolism. Ten PCs showed differential responses to mechanical wounding. These metabolites were classified as flavonoids that are usually associated with plant defense. In our study, 3,4-hydroxybenzoic acid, syringic acid, apigenin, and myricetin were significantly accumulated after mechanical treatment. Among these, 3,4-hydroxybenzoic acid was markedly responsive to the mechanical stress at 5 h across all treatment groups. 3,4-hydroxybenzoic acid belongs to C6C1-structure metabolites, and plays an important role in protecting nerves (Ju et al., 2015). Syringic acid with C6C3-structure metabolites can achieve sedative or anesthetic effects through central inhibition (Ogut, Armagan & Gül, 2022), and was massively accumulated after stimulation in the WUL treatment group. Our results showed that PCs with C6C3C6-structures were actively distributed and responded to mechanical stress. Apigenin and myricetin with C6C3C6-structure metabolites also have important medical value and were stimulated in the WLL group for 1 h and 3 h, respectively. Apigenin can inhibit the activity of carcinogens (Pang et al., 2021). Myricetin has a hypoglycemic effect (Negri et al., 2022). When accumulated, these metabolites will benefit plant defense.

After mechanical wounding, plants invest their resources to maximize their fitness (Impa et al., 2019). As shown here, to handle mechanical wounding, the metabolites of *C. roseus* were relocated in leaves and a new balance was established (Fig. 5). We propose that this relocation is the result of a source–sink relationship and is not correlated to organ biomass. In the WUL group, wounded upper leaves become a stronger sink relative to upper leaves and required more of an investment from the source. This would cause more response metabolites to be produced. Our results has shown that more sugars, TIAs, and parts of PCs were significantly accumulated in wounded upper leaves in the WUL group. By contrast, in the WLL group, upper leaves become a source and provided support for wounded lower leaves. However, the upper leaves were not fully mature and could not produce more comprehensive response metabolites. This led to more TCA compounds and fewer secondary metabolites being produced. The source–sink model provides a mechanism to regulate the resource distribution and effectively alter plant response patterns. This change directly led to a significant increase in the WLL group and a slow response to mechanical pressure. Therefore, more secondary metabolites were obtained without external influence.

## Conclusion

Changes in the source–sink relationship indicate that local responses in upper leaves have different effects among the CK, WUL, and WLL groups. Upper leaves of *C. roseus* in the WUL group required more substrates and energy to stimulate the production of secondary metabolites for defense. Conversely, in the WLL group, most secondary metabolites in the upper leaves were transported to the damaged region. These different response strategies resulted in discrepant synthetic pathways and secondary metabolite accumulation. Therefore, an observed increase in TIAs and/or PCs in response to stress may encourage a reevaluation of the commercial plant production practice to increase the yield of specific secondary metabolites for their usage in healthcare. In our study, the synthesis of TIAs stays at the upstream product and the content of our expected end products was low. In the future, we will pay more attention to the method of producing TIA end products.

##  Supplemental Information

10.7717/peerj.14539/supp-1 Supplemental Information 1 Raw data

## Additional Information and Declarations

### Data Availability

The following information was supplied regarding data availability: The raw data is available in the Supplementary File
